# Mixtures of Urinary Phenol and Phthalate Metabolite Concentrations in Relation to Serum Lipid Levels among Pregnant Women: Results from the EARTH Study

**DOI:** 10.3390/toxics12080574

**Published:** 2024-08-07

**Authors:** Xilin Shen, Maximilien Génard-Walton, Paige L. Williams, Tamarra James-Todd, Jennifer B. Ford, Kathryn M. Rexrode, Antonia M. Calafat, Dan Zhang, Jorge E. Chavarro, Russ Hauser, Lidia Mínguez-Alarcón, the EARTH Study Team

**Affiliations:** 1 Key Laboratory of Reproductive Genetics (Ministry of Education) and Department of Reproductive Endocrinology, Women’s Hospital, Zhejiang University School of Medicine, Hangzhou 310006, China; xilinshen@zju.edu.cn (X.S.); zhangdan@zju.edu.cn (D.Z.); 2 Department of Nutrition, Harvard T.H. Chan School of Public Health, Boston, MA 02115, USA; 3 Université Rennes, Inserm, EHESP, Irset (Institut de Recherche en Santé, Environnement et Travail)—UMR_S 1085, F-35000 Rennes, France; maximilien.genard-walton@univ-rennes.fr; 4 Department of Biostatistics and Epidemiology, Harvard T.H. Chan School of Public Health, Boston, MA 02115, USA; paige@sdac.harvard.edu; 5 Department of Epidemiology and Environmental Health, Harvard T.H. Chan School of Public Health, Boston, MA 02115, USA; tjtodd@hsph.harvard.edu (T.J.-T.); rhauser@hsph.harvard.edu (R.H.); 6 Department of Environmental Health, Harvard T.H. Chan School of Public Health, Boston, MA 02115, USA; jford@hsph.harvard.edu; 7 Division of Women’s Health, Department of Medicine, Brigham and Women’s Hospital, Harvard Medical School, Boston, MA 02115, USA; krexrode@bwh.harvard.edu; 8 National Center for Environmental Health, Centers for Disease Control and Prevention, Atlanta, GA 30329, USA; aic7@cdc.gov; 9 Clinical Research Center on Children’s Health of Zhejiang Province, Hangzhou 310006, China; 10 Departments of Nutrition and Epidemiology, Harvard T.H. Chan School of Public Health, Boston, MA 02115, USA; jchavarr@hsph.harvard.edu; 11 Channing Division of Network Medicine, Harvard Medical School & Brigham and Women’s Hospital, Boston, MA 02115, USA; 12 Department of Obstetrics, Gynaecology and Reproductive Biology, Harvard Medical School, Boston, MA 02115, USA

**Keywords:** mixture, endocrine disruptors, EDC, lipid, pregnancy

## Abstract

We examined whether mixtures of urinary concentrations of bisphenol A (BPA), parabens and phthalate metabolites were associated with serum lipid levels among 175 pregnant women who enrolled in the Environment and Reproductive Health (EARTH) Study (2005–2017), including triglycerides, total cholesterol, high-density lipoprotein (HDL), non-HDL, and low-density lipoprotein (LDL). We applied Bayesian Kernel Machine Regression (BKMR) and quantile g-computation while adjusting for confounders. In the BKMR models, we found no associations between chemical mixture and lipid levels, e.g., total cholesterol [mean difference (95% CRI, credible interval) = 0.02 (−0.31, 0.34)] and LDL [mean difference (95% CRI) = 0.10 (−0.22, 0.43)], when comparing concentrations at the 75th to the 25th percentile. When stratified by BMI, we found suggestive positive relationships between urinary propylparaben and total cholesterol and LDL among women with high BMI [mean difference (95% CRI) = 0.25 (−0.26, 0.75) and 0.35 (−0.25, 0.95)], but not with low BMI [mean difference (95% CRI) = 0.00 (−0.06, 0.07) and 0.00 (−0.07, 0.07)]. No association was found by quantile g-computation. This exploratory study suggests mixtures of phenol and phthalate metabolites were not associated with serum lipid levels during pregnancy, while there were some suggestive associations for certain BMI subgroups. Larger longitudinal studies with multiple assessments of both exposure and outcome are needed to corroborate these novel findings.

## 1. Introduction

Endocrine-disrupting chemicals (EDCs) are exogenous chemicals that can interfere with hormone action, and some are widely used in household products [1]. Specifically, di(2-ethylhexyl) phthalate (DEHP) is widely used in plasticizers, making plastics softer and more flexible, and can be found in many household items and medical devices, such as wall and floor coverings, food packaging, and medical tubing. DEHP is further metabolized into mono(2-ethylhexyl) phthalate (MEHP), mono(2-ethyl-5-hydroxyhexyl) phthalate (MEHHP), mono(2-ethyl-5-oxohexyl) phthalate (MEOHP), and mono(2-ethyl-5-carboxypentyl) phthalate (MECPP) in humans. Other low-molecular-weight phthalate metabolites such as monoethyl phthalate (MEP), mono-n-butyl phthalate (MBP) and monobenzyl phthalate (MBzP) are mainly found in people after exposure to personal care products (such as lotions and deodorants) and other consumer products [2,3]. Bisphenol A (BPA) is commonly used in polycarbonate plastics, epoxy resin in canned food liners, certain dental sealants and thermal receipts [4]. Parabens are used as preservatives in cosmetics, personal care products, pharmaceuticals, and food [5]. These EDCs can be detected and quantified in urine samples collected from almost all individuals in the industrialized world [6,7].

The adverse effects of maternal EDC exposure during pregnancy on the offspring are well-studied [8,9,10,11,12,13]. Pregnancy is a potentially sensitive period in relation to EDC exposure, with increased susceptibility to dyslipidemia and cardiovascular disease (CVD) [14]. During pregnancy, the body undergoes rapid cardiovascular and metabolic changes to adapt to the energy needs of the mother and the fetus [15]. Dyslipidemia is a well-known CVD risk factor characterized by high levels of triglycerides, total cholesterol and low-density lipoprotein (LDL), as well as lower levels of high-density lipoprotein (HDL), in the circulation [16]. Dyslipidemia during pregnancy has been associated with adverse fetal development [17,18], short- and long-term CVD risk [19,20,21] and higher BMI in the offspring [22]. It was reported in mice that high cholesterol levels during pregnancy impaired long-term vascular function in the mother [23], which supports the hypothesis that gestational dyslipidemia contributes to cardiovascular disorders later in life. Therefore, it is important to study the impact of EDCs during pregnancy, and the biomarkers and predictors of pregnancy-related and long-term cardiovascular health.

Some phthalates, parabens and BPA have been identified as metabolic disruptors [24,25], which contribute to metabolic disorders such as type 2 diabetes, fatty liver disease and metabolic syndrome. Some have been considered obesogens [26], which are defined as xenobiotic chemicals that can disrupt adipogenesis and energy balance [27]. Previously, in single-chemical analyses, we have found associations between lipid profiles during pregnancy and urinary phthalate metabolites [28], as well as parabens and phenols [29]. In real life, we are exposed to a mixture of EDCs, which may be associated with cholesterol and increase susceptibility to CVD. Interactions between these EDCs are biologically plausible, because previous experimental studies have demonstrated that exposure to parabens, phthalates and BPA can affect adipogenesis, as these chemicals can bind to peroxisome proliferator-activated receptors (PPARs) [30], which are present in adipose tissue and are key regulators of lipid metabolism [31,32]. When studying exposure to EDCs during pregnancy and its health effects, modern statistical tools are now available to evaluate their effects as a mixture [33].

Based on the metabolic-disrupting and obesogenic effects of phenols and phthalates, and our previous single-pollutant results, we hypothesized that among these pregnant women, mixtures of phenols and phthalate metabolite biomarkers would be related to serum lipid levels reflecting dyslipidemia. We also hypothesized that the associations would be stronger among women with overweight or obesity based on the presence of PPAR-γ in adipose tissues. Given the limited evidence related to EDC mixture and lipid profiles in pregnant women [34], we examined whether pregnancy mixtures of urinary concentrations of BPA, parabens and phthalate metabolites were associated with serum cholesterol levels among women who participated in the Environment and Reproductive Health (EARTH) Study. We further analyzed two subpopulations stratified by body mass index (BMI) to examine these associations.

## 2. Materials and Methods

### 2.1. Study Population

This study evaluated a subset of women enrolled in the Environment and Reproductive Health (EARTH) Study, a prospective cohort at the Massachusetts General Hospital (MGH) Fertility Center established to assess environmental and dietary determinants of fertility [35]. Women using their own gametes for fertility treatment between 18 and 45 years old were eligible to participate and approximately 60% of those contacted by the research staff were enrolled. This exploratory study includes 175 women enrolled from 2005 to 2017 who have data on both urinary concentrations of phenol and phthalate metabolite biomarkers as well as serum lipid biomarker measurements during pregnancy. The urine samples and blood samples were collected on the same day. Participants’ date of birth was collected at enrollment and their weight and height were measured by experienced study staff. Sociodemographic, lifestyle, and medical history questionnaires were administered to participants at the same entry visit. Study participants completed a comprehensive questionnaire on medical, family and reproductive history, use of consumer products, physical activity and smoking history. Infertility diagnosis under the Society of Assisted Reproductive Technology definitions (SART) was assigned by physicians [36]. Pregnancy-related covariates were abstracted from electronic medical records by trained study staff. The study obtained approvals from the Human Subject Committees of the Harvard T.H. Chan School of Public Health, MGH, and the Centers for Disease Control and Prevention (CDC). Informed consent was signed by participants after the study procedures had been explained, and all questions were answered in detail by trained staff.

### 2.2. Exposure Assessment

Enrolled women provided urine samples at the clinic during pregnancy. For this study, we included the urine sample that was collected on the same day the blood sample was collected at the clinic visit during pregnancy. The specific gravity of the urine was measured by a handheld refractometer (National Instrument Company, Inc., Baltimore, MD, USA) at room temperature, calibrated with deionized water before each measurement. We included specific gravity as a covariate in the statistical models as previously described [37,38]. Urine samples were stored at −80 °C after collection and then shipped frozen to the CDC for analysis overnight. As previously described [39,40], we applied strict quality controls and used online solid-phase extraction along with isotope dilution–high-performance liquid chromatography–tandem mass spectrometry to quantify the concentrations of phenol and phthalate biomarkers in the urine samples collected. The measured chemicals included four phenols (BPA, methylparaben, propylparaben and butylparaben) four phthalate metabolites [mono-n-butyl phthalate (MBP), mono-isobutyl phthalate (MiBP), monoethyl phthalate (MEP) and monobenzyl phthalate (MBzP)] and four DEHP phthalate metabolites [mono(2-ethylhexyl) phthalate (MEHP), mono(2-ethyl-5-hydroxyhexyl) phthalate (MEHHP), mono(2-ethyl-5-oxohexyl) phthalate (MEOHP) and mono(2-ethyl-5-carboxypentyl) phthalate (MECPP)]. The above twelve biomarkers have been examined as a mixture in this study. Limits of detection (LODs) ranged from 0.2 to 1.2 µg/L, depending on the chemical biomarkers (Supplemental Table S1). Quality control was performed as previously described using statistical probability rules [41].

### 2.3. Outcome Assessment

We included one non-fasting blood sample per participant collected on the same day as the urine sample. If the participant provided several samples from different timepoints during pregnancy, we randomly selected one sample at one timepoint. Blood samples were prepared as previously described [29] and transferred to the Clinical and Epidemiologic Laboratory (CERLab) at Boston Children’s Hospital (Boston, MA, USA), which was certified by the Centers for Disease Control and Prevention/National Heart, Lung, and Blood Institute Lipid Standardization Program. Total cholesterol, triglycerides and HDL cholesterol levels (mg/dL) were measured in the serum sample with the Roche Cobas 6000 system, and reagents and calibrators were provided by Roche Diagnostics (Indianapolis, IN, USA), which were approved by the Food and Drug Administration (FDA) for clinical use.

Triglycerides were measured enzymatically with correction for endogenous glycerol, as previously described [42]. Cholesterol levels were also measured enzymatically [43]. The concentrations of HDL-C were measured by a direct enzymatic colorimetric assay, which meets the rigid requirements established by the Lipid Standardization Program [44]. Triglyceride concentrations were determined with an intra- and inter- day-to-day reproducibility of 1.8% and 1.7%, respectively, and the corresponding figures for HDL-C levels were 3.3% and 1.7%. The coefficients of variation (CVs) for total cholesterol concentrations were 1.7% and 1.6%. Non-HDL levels were calculated as the difference between total and HDL cholesterol concentrations. LDL cholesterol was estimated using the Friedewald formula [45].

### 2.4. Statistical Analysis

The demographic, reproductive characteristics of the study population and serum lipid biomarker levels were described as median ± inter-quartile ranges (IQRs) for continuous variables and count (percentage) for discrete variables. The serum lipid biomarker levels were not log-transformed because they appeared to be normally distributed [28] based on Kolmogorov–Smirnov tests for normality. Distributions of urinary concentrations of chemical biomarkers were reported using percentiles, geometric mean and mean ± standard deviations (SDs). Detection frequencies of the urinary chemical biomarkers were reported. Given that the samples were analyzed in multiple batches over the years, some biomarkers had different LODs at various time points; for those biomarkers, we reported the maximum LOD (Supplemental Table S1). Concentrations below the LODs were imputed using R package multiLODmice (version 0.1.0), which extended the multiple imputation method to allow different LODs for different observations [46]. Correlations between urinary chemical biomarkers were estimated using Spearman correlation coefficients. All urinary chemical biomarkers were log-transformed by a natural logarithm before including them in the statistical models.

Covariates were selected based on prior knowledge regarding their impact on exposures and outcomes. Primary models were adjusted for urine specific gravity, age (years), pre-pregnancy BMI (kg/m^2^) at sample collection, race (white/Caucasian and other, combined given the relatively low proportion of women of color included in this study), education level (graduate degree attainment), infertility diagnosis by physician (female factor; male factor; unexplained cause), mode of conception [without treatment; use of in vitro fertilization (IVF)/intrauterine insemination (IUI)], multiple gestations (singleton; twins/triplets) and trimester at sample collection (1st; 2nd; 3rd). In stratified models, the pre-pregnancy BMI was not adjusted. Mixture effect analyses were performed using two modern approaches: Bayesian Kernel Machine Regression (BKMR) [47] and quantile g-computation [48]. The BKMR method aims to model the complex relationship between a number of variables and the outcome (dependent variable) using the flexible non-linear or additive function $h\left(\cdot \right)$. The general modeling framework considered is:

$$g\left(\mu_{i}\right)\sim h\left(z_{i1},\cdots ,z_{iM}\right)+\beta x_{i},i=1,\cdots ,n$$

where g is the monotonic link function, $\mu_{i}=E(Y_{i})$, h is the flexible function of predictors (exposure variables $z_{i1},\cdots ,z_{iM}$), x is a vector of covariates considered to have linear relationships with the outcome, and β is the corresponding coefficients for each sample i in a total of n samples [47].

In the BKMR analysis, to account for the collinearity of biomarker concentrations, hierarchical variable selection was applied, which means biomarker concentrations were categorized into non-overlapping groups, and variable selection was performed first at the group level and then within groups [47]. We grouped the chemical biomarkers based on prior knowledge of their sources, their correlations, and previous findings in the same cohort: (1) BPA and DEHP metabolites (MEHP, MEHHP, MEOHP and MECPP); (2) parabens (methylparaben, propylparaben, butylparaben); (3) other phthalate metabolites (MBP, MiBP, MEP, MBzP). In each analysis, the number of iterations was 20,000. We reported group-specific posterior inclusion probabilities (PIPs) and conditional posterior inclusion probabilities, which represent the group contribution and the chemical biomarker’s individual contribution to the non-null association between exposure and the outcome.

In secondary analyses, we also evaluated stratification by BMI (above and below 25 kg/m^2^), which is the threshold for overweight and obesity [49], and studied chemicals individually and as the molar sums of the chemicals within each group (only when applying BKMR). We illustrated graphically for each chemical: (1) exposure–response relationships, while holding all other biomarkers at median concentrations, and (2) the mean difference between the 75th and 25th percentiles (estimates and 95% credible intervals) when concentrations of all other biomarker were held at the 25th, 50th, and 75th.

In the quantile g-computation models, we reported the mixture effect by the mean differences and 95% confidence intervals for each outcome. All analyses were performed in R environment (version 4.0.5). BKMR analyses were conducted using the R package bkmr (version 0.2.2) and quantile g-computation analyses were conducted using the R package qgcomp (version 2.15.2).

## 3. Results

The 175 women included in this exploratory study had a median (IQR) age of 35 (32, 38) years at sample collection during pregnancy and a pre-pregnancy BMI of 22.9 (21.2, 25.6) kg/m^2^ (Table 1). Thirty percent of the participants (N = 53) had overweight or obesity. The majority of the cohort were white (88%) and highly educated (60% possessed a graduate degree), and few were current or past smokers (29%). Of the women included in this study, 83% became pregnant after medical intervention, with 57% using IVF and 26% using IUI. Most women had singleton pregnancies (82%). Compared to women with no exposure and outcome assessment, the included women were more likely to undergo IVF treatments and diagnosed female-factor-caused infertility [22,23]. The median (IQR) serum concentrations of total triglycerides, total cholesterol, HDL, non-HDL and LDL cholesterol were 181 (112, 251), 229 (190, 279), 68 (58, 79), 161 (122, 204) and 120 (92, 158) mg/dL, respectively.

The detection frequencies for BPA (87%), butylparaben (58%) and MEHP (69%) were lower than for other chemical biomarkers (≥93%) (Supplemental Table S1). Compared to adult females participating in the National Health and Nutrition Examination Survey (NHANES) [6], women in this study had similar urinary chemical biomarker concentrations, except for lower concentrations of MBzP and higher concentrations of propylparaben. Urinary concentrations of the four DEHP metabolites (MEOHP, MEHHP, MECPP, MEHP) were highly correlated (Spearman r range from 0.76 to 0.98, Supplemental Figure S1). DEHP metabolites were moderately correlated with BPA (Spearman r range from 0.37 to 0.47). In addition, concentrations of methylparaben and propylparaben were highly correlated (Spearman r = 0.86). Butylparaben was weakly correlated with other chemicals (Spearman r ≤ 0.31).

**Figure 1:**
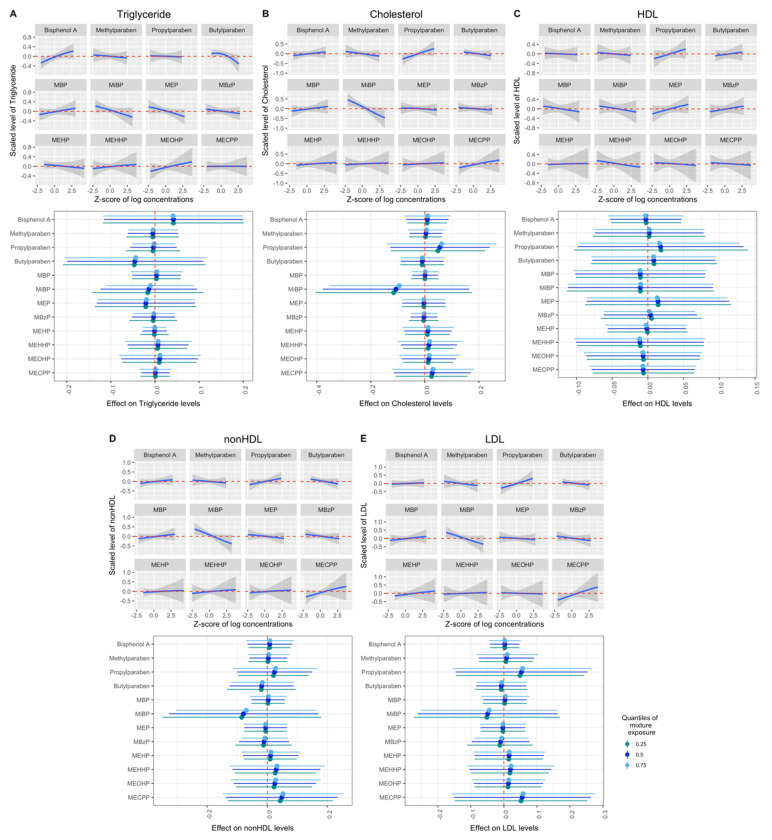
Bayesian Kernel Machine Regression (BKMR) mixture associations of phenol and phthalate metabolites and lipid biomarkers. (**A**–**E**) Mixture associations with total triglycerides, total cholesterol, HDL, non-HDL and LDL cholesterol in BKMR. Upper half: exposure–response relationships for each biomarker while holding all other biomarkers at their medians. Lower half: mean difference between the 75th and 25th percentiles of exposure (estimates and 95% credible intervals) when other biomarker concentrations were fixed at the 25th, 50th, and 75th percentiles. BKMR models were adjusted for age, education level, race, infertility diagnosis, mode of conception, number of fetuses, trimester and specific gravity. HDL: high-density lipoprotein. LDL: low-density lipoprotein. MEHP: mono(2-ethylhexyl) phthalate. MEHHP: mono(2-ethyl-5-hydroxyhexyl) phthalate. MEOHP: mono(2-ethyl-5-oxohexyl) phthalate. MECPP: mono(2-ethyl-5-carboxypentyl). MBP: mono-n-butyl phthalate. MiBP: mono-isobutyl phthalate. MEP: monoethyl phthalate. MBzP: monobenzyl phthalate.

The primary BKMR models showed no significant overall mixture or single-pollutant effects when comparing mixed-exposure biomarker concentrations at the 75th to the 25th percentile on total triglycerides (mean difference = −0.03, 95% CRI = −0.29, 0.23), total cholesterol (mean difference = 0.02, 95% CRI = −0.31, 0.34), HDL (mean difference = 0.00, 95% CRI = −0.27, 0.26), non-HDL (mean difference = 0.04, 95% CRI = −0.27, 0.35), or LDL (mean difference = 0.10, 95% CRI = −0.22, 0.43) cholesterol (Figure 1), although PIPs were greater than 50% in some models (Supplemental Table S2). In addition, no significant interactions between chemical biomarkers were observed (Figure 1). In addition, no associations were found between a quartile increase in the chemical biomarker mixture and total triglycerides using quantile g-computation (mean difference = −0.11, 95% CRI = −0.32, 0.09), total cholesterol (mean difference = 0.04, 95% CRI = −0.17, 0.26), HDL (mean difference = 0.05, 95% CRI = −0.24, 0.34), non-HDL (mean difference = 0.03, 95% CRI = −0.18, 0.25) or LDL (mean difference = 0.09, 95% CRI = −0.17, 0.34) (Supplemental Table S3).

**Figure 2:**
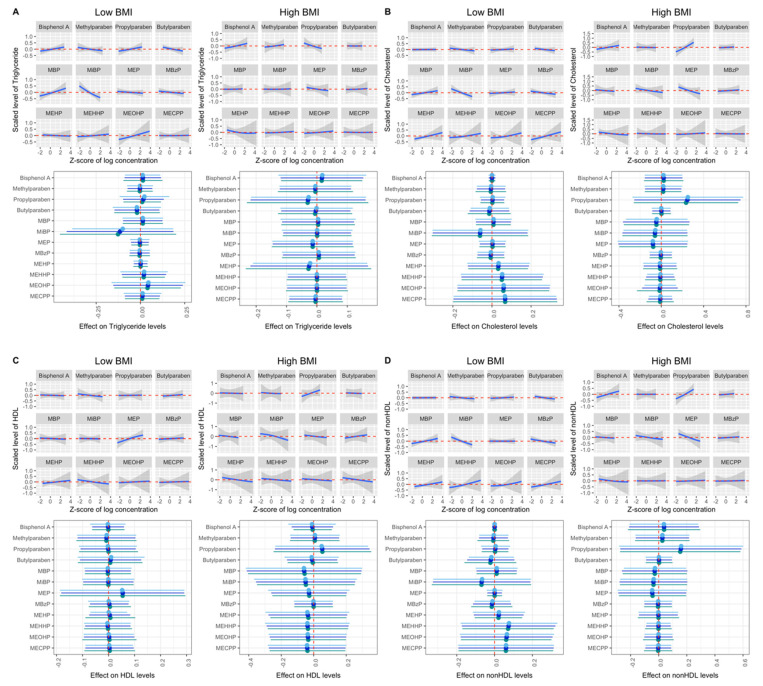

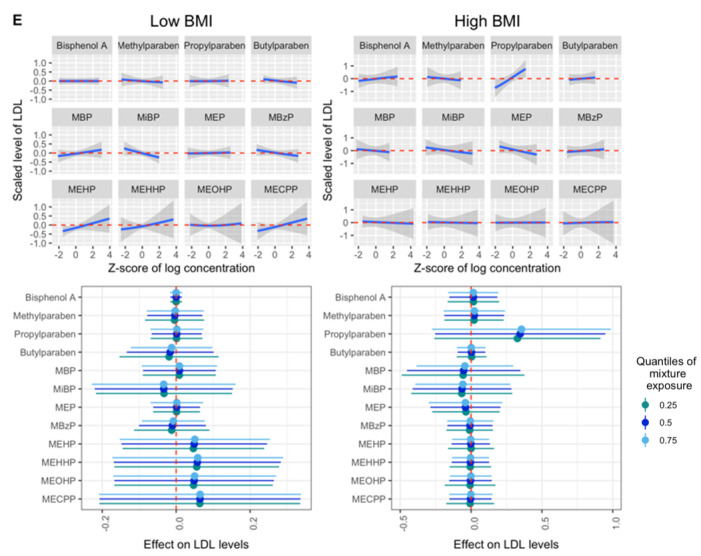
Bayesian Kernel Machine Regression (BKMR) mixture associations of phenol and phthalate metabolites and lipid biomarkers when stratified by BMI. (**A**–**E**) Mixture associations with total triglycerides, total cholesterol, HDL, non-HDL and LDL cholesterol in BKMR. Upper half: exposure–response relationships for each biomarker while holding all other biomarkers at their median. Lower half: mean difference between the 75th and 25th percentile of exposure (estimates and 95% credible intervals) when other biomarker concentrations were fixed at the 25th, 50th, and 75th percentiles. BKMR models were adjusted for age, education level, race, infertility diagnosis, mode of conception, number of fetuses, trimester and specific gravity. HDL: high-density lipoprotein. LDL: low-density lipoprotein. MEHP: mono(2-ethylhexyl) phthalate. MEHHP: mono(2-ethyl-5-hydroxyhexyl) phthalate. MEOHP: mono(2-ethyl-5-oxohexyl) phthalate. MECPP: mono(2-ethyl-5-carboxypentyl). MBP: mono-n-butyl phthalate. MiBP: mono-isobutyl phthalate. MEP: monoethyl phthalate. MBzP: monobenzyl phthalate.

We then examined the associations between chemical biomarker mixtures and lipid profiles, stratifying by BMI. We observed suggestive positive relationships of urinary propylparaben with total cholesterol and LDL among women with high BMI [mean difference (95% CRI) = 0.25 (−0.26, 0.75) and 0.35 (−0.25, 0.95), conditional PIPs = 79.0% and 83.2%, Figure 2 and Supplemental Table S4] when comparing concentrations at the 75th to the 25th percentile, fixing other chemicals at their medians. The corresponding figures of urinary propylparaben with total cholesterol and LDL were 0.00 (−0.06, 0.06) and 0.00 (−0.07, 0.07), with conditional PIPs of 24.8% and 25.5% among women with low BMI. While we observed different estimates for women with high and low BMI, the differences were not statistically significant. The associations between mixtures of these EDCs and lipid profiles did not differ between high- and low-BMI groups when applying quantile g-computation (Supplemental Table S5).

## 4. Discussion

In this exploratory study, we examined whether mixtures of urinary concentrations of four phenol and eight phthalate metabolites were associated with serum lipid levels during pregnancy, including total triglycerides, total cholesterol, HDL, non-HDL and LDL cholesterol, among pregnant women in the EARTH Study cohort. The mixture models were evaluated using BKMR modeling and quantile g-computation. We observed no overall associations of urinary phenol and phthalate metabolite biomarker mixtures with lipid biomarkers in both models. Nevertheless, when stratified by BMI, we found suggestive positive relationships between propylparaben, total cholesterol and LDL among women with high BMI. It is important to note that these relationships were not statistically significant, possibly due to the moderate sample size included in the study, which limited our study power. No difference in mixture effect was observed using quantile q-computation models among women with high and low BMI.

To the best of our knowledge, only one cohort study examined the mixture effect of EDCs on circulating lipid levels in pregnant women [34]. The authors evaluated BPA, phthalates, polybrominated diphenyl ethers (PBDEs), and per- and polyfluoroalkyl substances (PFAS), but not parabens. No overall mixture associations with the examined lipids were reported, either. However, they identified urinary MBzP, measured at 16 weeks of pregnancy, as an important contributor to triglycerides levels. Other individual phthalate biomarkers and BPA were weak contributors to the association with total lipid, cholesterol and triglycerides levels, which is consistent with our results. In our study, we found no associations between the urinary concentration of MBzP and triglyceride levels. This may be partially explained by the fact that the pregnant women in our study had considerably lower urinary MBzP concentrations compared to women in the HOME Study (75th quantile: 5.75 μg/L vs. 24.5 μg/L). Another reason may be related to the strategy used when analyzing chemical biomarker exposure mixtures, as our primary analyses applied hierarchical variable selection in BKMR and included the exposure biomarkers as groups based on their correlation and previous publications in EARTH. Given the scarce literature on the topic in relation to pregnant women, additional studies are warranted.

Previously, in single-pollutant analyses among the same women in the EARTH Study, it was found that urinary propylparaben concentrations were positively associated with total cholesterol, as well as non-HDL and LDL cholesterol, levels when comparing concentrations in the highest tertile to concentrations in the lowest [29]. They also found several associations between urinary MBP, BPA and several DEHP metabolites and lipid levels when comparing the concentrations in the highest group versus the lowest group [28]. In single-biomarker analyses, urinary propylparaben was associated with total, non-HDL and LDL cholesterol. In the previous study, urinary concentrations of pollutants were evaluated as categorical variables, and statistical testing was performed against the lowest quantiles, whereas in this mixture manuscript, urinary biomarker concentrations were examined as continuous variables within a determined group. Some of the previously observed positive associations may also be neutralized by the negative effects of other EDC biomarkers. For example, there is a decreasing trend in the association between MiBP and total cholesterol, non-HDL and LDL (Figure 1). The moderate sample size included in the study also limited our study’s ability to find significant associations by BKMR. This may explain the discrepancy between single and mixture model results for the other examined biomarkers.

We observed suggestive positive relationships of propylparaben with total cholesterol and LDL among women with high BMI scores. Some parabens were identified as obesogens [26]. Wen and colleagues found positive associations between urinary paraben concentrations, including propylparaben and mixtures of parabens, and gestational weight gain among pregnant women in Wuhan, China [50]. They also observed that these associations were stronger among women with overweight or obesity compared to women with normal- and underweight.

Exposure to parabens might result in elevated circulating lipid levels by activating the peroxisome proliferator-activated receptor (PPAR)-γ [30], which is presented mainly in adipose tissue and key regulators in the lipid metabolism [31,51]. In addition, parabens were found in adipose tissue [52]. Thus, women with overweight and obesity might be more sensitive to paraben exposure than others. Particularly, urinary propylparaben showed a suggestive positive relationship with total cholesterol and LDL among women with high BMI, and was the main contributor. Women in our study had higher concentrations of parabens compared to the Wuhan cohort, and higher concentrations of propylparaben compared to adult females participating in NHANES, while they had similar concentrations of other parabens [6]. This fact may explain why we observed a stronger relationship with propylparaben in single-pollutant analyses in BKMR, but not with the other parabens among women with higher BMI. However, additional studies on both animal models and humans are required to further understand the relationship between BMI, parabens and circulating lipid profile.

There are some limitations to this study that should be noted. First, the generalizability of the results may be limited as this study includes subfertile women seeking treatment in fertility clinics. However, these women are also at higher risk of CVD compared to women in the general population [53]. Second, we cannot confirm that all serum samples were collected after fasting, and this may affect the results. Third, lipid levels physiologically rise during pregnancy, and adjusting for trimester may not fully account for this change. Fourth, the sample sizes after stratification by high (N = 53) and low BMI (N = 122) were relatively low, which may limit our ability to detect significant associations. Fifth, exposure misclassification is possible considering the biomarkers’ short biological half-lives [3] and the episodic nature of exposures [54], especially since we used one urine sample per study participant. However, in the context of EARTH, we have previously reported the moderate to high correlations of chemical biomarkers measured across multiple urine samples in women demonstrating low variability [55]. Lastly, as a cross-sectional analysis, reverse causation is also possible. Residual confounding by other exposures, lifestyle and nutritional factors is possible.

Despite the above limitations, our study has several strengths. Our study is one of the first studies to evaluate mixtures of BPA, parabens and phthalate metabolites simultaneously in pregnant women. A major strength of this study is that we applied two sophisticated statistical methods to estimate associations of phenol and phthalate biomarkers with outcomes of interest as a mixture: BKMR and quantile g-computation. Quantile g-computation assumes linearity and additivity on the quantile scale of the chemical biomarkers’ concentrations, and estimates the association between a quantile increase in all exposed chemical biomarkers simultaneously and the outcome [48]. The study is interpretable and efficient, in line with the conventional mode of comparison among quantiles. BKMR, on the other hand, allows for non-additive interactions between chemical biomarkers and complex non-linear exposure–response relationships [47]. This modeling method is flexible and powerful, yet not that interpretable or efficient compared to quantile g-computation. Furthermore, hierarchical variable selection was applied when taking highly correlated pollutants and pollutants with the same sources, such as DEHP metabolites, into account. This grouping method utilized existing knowledge of co-exposure and observed correlations, enabling the more precise evaluation of individual relevance. Previously, approaches modeling EDC exposures as a mixture have been successfully applied in reproductive epidemiology [56,57,58,59], and provided valuable insights for their joint effects on glucose metabolism during pregnancy, ovarian reserves, lipid profiles in adult women, and risk of infertility. Applying such sophisticated statistical methods will help us better understand the effects of mixtures of EDCs on human health. Other strengths include the comprehensive adjustments for other reproductive and demographical confounders, as well as the evaluation of effects on lipid profiles in a well-established sub-fertile cohort with higher CVD risk, who may be particularly vulnerable to the impacts of EDCs as metabolic disruptors.

## 5. Conclusions

In summary, we observed overall no association between a mixture of BPA, parabens and phthalate metabolites and circulating lipid levels, including total triglycerides, total cholesterol, and HDL, non-HDL and LDL cholesterol, among 175 pregnant women. However, we found suggestive positive associations between urinary propylparaben, total cholesterol and LDL among women with high BMI. This study suggests there is no association between the mixture of phenols and phthalate metabolites and serum lipid levels among pregnant women, while there are some suggestive associations for certain BMI subgroups. Larger longitudinal studies with multiple assessments of both exposure and outcome are needed to corroborate these novel findings. These results, if confirmed, add to our knowledge of the effects of exposure to multiple EDCs and pregnancy health.

**Note:** The findings and conclusions in this report are those of the authors and do not necessarily represent the official position of the Centers for Disease Control and Prevention (CDC). Use of trade names is for identification purposes only and does not imply endorsement by the CDC, the Public Health Service, or the U.S. Department of Health and Human Services.

## Figures and Tables

**Table 1:** Demographics, reproductive characteristics and serum lipid profiles among 175 pregnant women enrolled in the Environment and Reproductive Health (EARTH) Study.

Demographic Characteristics	
Age at pregnancy, years, median (IQR)	35 (32, 38)
Race, N (%)	
White	154 (88)
Black	5 (2)
Asian	8 (5)
Other	8 (5)
Pre-pregnancy body mass index, kg/m^2^	22.9 (21.2, 25.6)
<25, N (%)	122 (70)
≥25, N (%)	53 (30)
Ever smoked, N (%)	50 (29)
Graduate degree attainment, N (%)	105 (60)
Primary infertility diagnosis, N (%)	
Male factor	58 (33)
Female factor	59 (33)
Unexplained	58 (33)
Mode of conception, N (%)	
IUI	45 (26)
IVF	101 (57)
Natural	29 (17)
Number of babies, N (%)	
Singleton	144 (82)
Twins and triplets	31 (18)
Trimester of sample collection, N (%)	
1st	61 (35)
2nd	47 (27)
3rd	67 (38)
Serum lipid level, mg/dL, median (IQR)	
Total triglycerides	181 (112, 251)
Total cholesterol	229 (190, 279)
HDL cholesterol	68.0 (58.0, 79.0)
Non-HDL cholesterol	161 (122, 204)
LDL cholesterol	120 (92.0, 158)

N: Number of participants. IUI: intrauterine insemination. IVF: in vitro fertilization. HDL: high-density lipoprotein. LDL: low-density lipoprotein.

## Data Availability

The data are not publicly available due to privacy and confidentiality reasons.

## Supplementary Materials

The following supporting information can be downloaded at: https://www.mdpi.com/article/10.3390/toxics12080574/s1, Table S1: Distribution of urinary concentration (μg/L) of phenol and phthalate metabolites among pregnant women in Environment and Reproductive Health (EARTH) Study (2005–2017); Table S2: Bayesian Kernel Machine Regression (BKMR) posterior inclusion probabilities (PIPs) (%) of phenol and phthalate metabolites in relation to serum lipid biomarkers; Table S3: Quantile g-computation measurement of the association between a quartile increase in mixture chemical biomarker concentrations and lipid biomarkers; Table S4: Bayesian Kernel Machine Regression (BKMR) posterior inclusion probabilities (PIPs) (%) for chemicals in relation to lipid profiles stratified by high vs. low BMI; Table S5: Quantile g-computation measurement of the association between a quartile increase in mixture chemical biomarker concentrations and lipid biomarkers stratified by high vs. low BMI; Figure S1: Spearman correlation between pairs of phenol and phthalate metabolite concentrations.
