# Supplementary material for: Mixtures of Urinary Phenol and Phthalate Metabolite Concentrations in Relation to Serum Lipid Levels among Pregnant Women: Results from the EARTH Study
Source: Toxics. 2024 Aug 7;12(8):574. doi: 10.3390/toxics12080574 (PMC11359712; doi:10.3390/toxics12080574)
Supplement: Supplementary file 1 [file toxics-12-00574-s001.zip › toxics-3088821-supplementary.pdf]

Supplementary Materials

Mixtures of urinary phenol and phthalate metabolite concentrations in relation to serum lipid levels among pregnant women: results from the EARTH Study.

Table of Contents

Supplemental Table S1. Distribution of urinary concentration (µg/L) of phenol and phthalate metabolites among pregnant women in Environment and Reproductive Health (EARTH) Study (2005-2017)... 2

Supplemental Figure S1. Spearman Correlation between pairs of phenol and phthalate metabolite concentrations......................... 4

Supplemental Table S2. Bayesian Kernel Machine Regression (BKMR) posterior inclusion probabilities (PIPs) (%) of phenol and phthalate metabolites in relation to serum lipid biomarkers... 5

Supplemental Table S3. Quantile g-computation measurement of the association between a quartile increase in mixture chemical biomarker concentrations and lipid biomarkers... 7

Supplemental Table S4. Bayesian Kernel Machine Regression (BKMR) posterior inclusion probabilities (PIPs) (%) of phenol and phthalate metabolites in relation to serum lipid biomarkers stratified by high vs low BMI.. 8

Supplemental Table S5. Quantile g-computation measurement of the association between a quartile increase in mixture chemical biomarker concentrations and lipid biomarkers stratified by high vs low BMI.. 10

Supplemental Table S1. Distribution of urinary concentration (µg/L) of phenol and phthalate metabolites among pregnant women in Environment and Reproductive Health (EARTH) Study (2005-2017).

	N	Detection Frequency, %	Maximum LOD (µg/L)	25th	Median	Geometric mean	SD	Mean	75th	95th
BPA	175	87	0.4	<mLOD	1.00	1.20	2.36	1.90	1.75	5.76
Methylparaben	175	99	1.0	25.8	73.9	94.8	640	333	364	1440
Propylparaben	175	98	0.2	4.00	23.2	19.5	211	88.6	105	357
Butylparaben	175	58	0.2	<mLOD	0.30	1.82	15.4	6.83	2.55	19.8
MBP	175	97	0.6	3.80	9.20	9.18	32.0	18.0	18.7	46.6
MiBP	175	98	0.8	2.45	6.10	5.79	15.5	10.1	12.2	30.5
MEP	175	100	1.2	11.7	31.3	39.6	544	200	108	1060
MBzP	175	93	0.2	0.9	2.50	3.08	17.2	8.13	5.75	24.8
MEHP	175	69	1.2	<mLOD	1.80	3.90	60.0	15.2	4.80	26.8
MEHHP	175	99	0.7	2.90	7.00	8.21	240	49.2	18.1	89.5
MEOHP	175	98	0.7	2.55	5.60	5.99	144	31.4	13.9	55.4
MECPP	175	100	0.4	5.60	11.7	13.4	254	59.1	27.9	102
ΣDEHP	175	-	-	0.04	0.09	0.10	2.28	0.50	0.22	0.91

N: number of participants; LOD: Limit of detection; mLOD: maximum LOD; SD: Standard derivation; BPA: bisphenol A; MBP: mono-n-butyl phthalate; MiBP: mono-isobutyl phthalate; MEP: monoethyl phthalate; MBzP: monobenzyl phthalate; MEHP: mono(2-ethylhexyl) phthalate; MEHHP: mono(2-ethyl-5-hydroxyhexyl) phthalate; MEOHP: mono(2-ethyl-5-oxohexyl) phthalate; MECPP: mono(2-ethyl-5-carboxypentyl) phthalate; ∑DEHP: molar sum of di(2-ethylhexyl) phthalate metabolites, including MEHP, MEHHP,

MEOHP and MECPP. Urinary concentrations are presented as unadjusted for urine dilution. There were multiple LODs for each biomarker and the maximum LODs were reported.

Supplemental Figure S1. Spearman Correlation between pairs of phenol and phthalate metabolite concentrations.

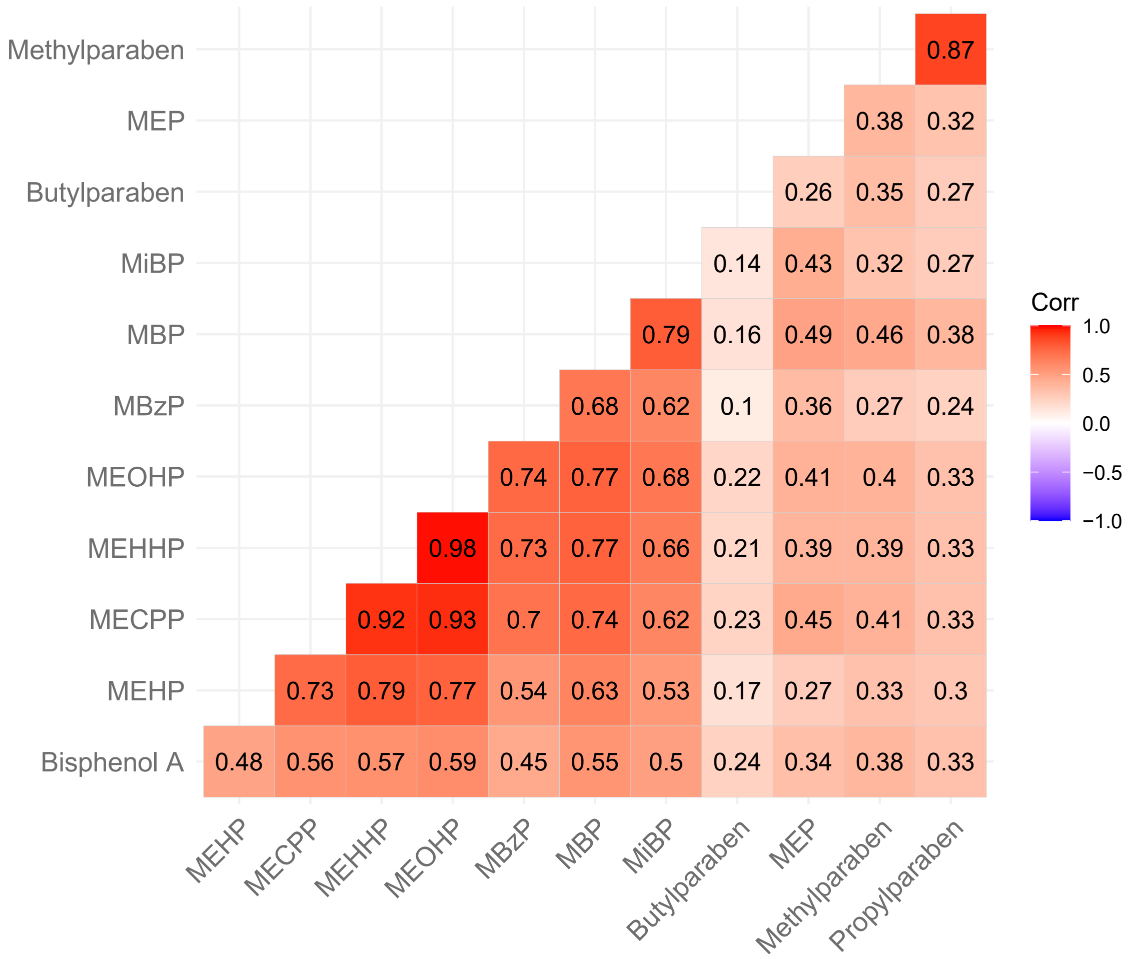

Supplemental Table S2. Bayesian Kernel Machine Regression (BKMR) posterior inclusion probabilities (PIPs) (%) of phenol and phthalate metabolites in relation to serum lipid biomarkers.

	Group	Triglyceride		Cholesterol		HDL		Non-HDL		LDL	
		groupPIP	condPIP	groupPIP	condPIP	groupPIP	condPIP	groupPIP	condPIP	groupPIP	condPIP
BPA	1	47.8	56.3	65.1	14.1	65.6	13.9	80.1	9.1	70.9	5.5
Methylparaben	2	59.6	10	61.1	16.9	58.3	29.3	49.7	20.6	51.6	20.5
Propylparaben	2	59.6	9.2	61.1	61.6	58.3	39.2	49.7	43.1	51.6	60.5
Butylparaben	2	59.6	80.8	61.1	21.5	58.3	31.5	49.7	36.3	51.6	19
MBP	3	39.5	14.1	67.7	7.7	63.8	25.3	62.7	9.4	50.4	15.5
MiBP	3	39.5	26.4	67.7	71.9	63.8	25.2	62.7	59	50.4	48.8
MEP	3	39.5	46.2	67.7	10.1	63.8	29.3	62.7	13.5	50.4	15.2
MBzP	3	39.5	13.3	67.7	10.3	63.8	20.2	62.7	18.1	50.4	20.5
MEHP	1	47.8	5.3	65.1	17.4	65.6	17.3	80.1	12	70.9	17.6
MEHHP	1	47.8	13.7	65.1	19.6	65.6	25.5	80.1	24.2	70.9	20.5
MEOHP	1	47.8	19	65.1	20.5	65.6	21.6	80.1	21.4	70.9	15.6
MECPP	1	47.8	5.8	65.1	28.4	65.6	21.7	80.1	33.3	70.9	40.8

groupPIP: group-specific posterior inclusion probability; condPIP: conditional posterior inclusion probability; BPA: bisphenol A; MBP: mono-n-butyl phthalate; MiBP: mono-isobutyl phthalate; MEP: monoethyl phthalate; MBzP: monobenzyl phthalate; MEHP: mono(2-ethylhexyl) phthalate; MEHHP: mono(2-ethyl-5-hydroxyhexyl) phthalate; MEOHP: mono(2-ethyl-5-oxohexyl) phthalate; MECPP: mono(2-ethyl-5-carboxypentyl) phthalate; HDL: high density lipoprotein; LDL: low-density lipoprotein.

BKMR model was adjusted for age at sample collection, pre-pregnancy body mass index (BMI), education level, race, infertility diagnosis, mode of conception, number of fetuses, trimester and specific gravity. Hierarchical variable selection was done by grouping DEHP metabolites and BPA, parabens and other phthalate metabolites.

Supplemental Table S3. Quantile g-computation measurement of the association between a quartile increase in mixture chemical biomarker concentrations and lipid biomarkers.

	Mean difference	95% Confidence Interval
Triglyceride	-0.11	-0.32 - 0.09
Cholesterol	0.04	-0.18 - 0.26
HDL	0.05	-0.24 - 0.34
Non-HDL	0.03	-0.19 - 0.25
LDL	0.08	-0.17 - 0.33

HDL: high-density lipoprotein; LDL: low-density lipoprotein.

Quantile g-computation model was adjusted for age at sample collection, pre-pregnancy body mass index, education level, race, infertility diagnosis, mode of conception, number of fetuses, trimester and specific gravity.

Supplemental Table S4. Bayesian Kernel Machine Regression (BKMR) posterior inclusion probabilities (PIPs) (%) of phenol and phthalate metabolites in relation to serum lipid biomarkers stratified by high vs low BMI.

	Group	Triglyceride		Cholesterol		HDL		Non-HDL		LDL	
		groupPIP	condPIP	groupPIP	condPIP	groupPIP	condPIP	groupPIP	condPIP	groupPIP	condPIP
High BMI											
BPA	1	50.7	23	50.3	25.6	70.9	10.4	49.4	32.2	50.6	24.2
Methylparaben	2	44.2	22.7	75.8	13.6	55.4	26.2	67.5	21.2	83.2	11.1
Propylparaben	2	44.2	49.5	75.8	79	55.4	41.7	67.5	67.3	83.2	83.2
Butylparaben	2	44.2	27.8	75.8	7.4	55.4	32.1	67.5	11.5	83.2	5.7
MBP	3	39.5	25.5	66.5	22.3	65.5	30.2	57.2	25.1	62.3	25.1
MiBP	3	39.5	22.2	66.5	28.6	65.5	30.7	57.2	27.5	62.3	31.3
MEP	3	39.5	29.8	66.5	39.4	65.5	23.7	57.2	34.5	62.3	25.9
MBzP	3	39.5	22.4	66.5	9.7	65.5	15.4	57.2	12.9	62.3	17.7
MEHP	1	50.7	29.2	50.3	20.3	70.9	21.5	49.4	18.9	50.6	17.2
MEHHP	1	50.7	14.5	50.3	16.2	70.9	21.4	49.4	14.8	50.6	18
MEOHP	1	50.7	15.3	50.3	18.9	70.9	21.8	49.4	15.4	50.6	18.8
MECPP	1	50.7	18	50.3	18.9	70.9	24.9	49.4	18.7	50.6	21.8
Low BMI											
BPA	1	58.8	15.5	91.1	0.7	41.3	16.7	92.4	0.5	91.8	0.6
Methylparaben	2	43	20.7	26	31.9	44.4	33.8	33.1	29.2	29.9	29
Propylparaben	2	43	37.7	26	24.8	44.4	31.3	33.1	26.5	29.9	25.5
Butylparaben	2	43	41.7	26	43.3	44.4	34.9	33.1	44.3	29.9	45.5
MBP	3	69.1	17.3	44	14.5	53.4	15.3	51	16.9	39.5	17.7
MiBP	3	69.1	69.8	44	57.8	53.4	17.9	51	55	39.5	45.4
MEP	3	69.1	5.8	44	13.7	53.4	49.7	51	6.4	39.5	13.2
MBzP	3	69.1	7.1	44	13.9	53.4	17.1	51	21.8	39.5	23.7
MEHP	1	58.8	7.4	91.1	19.3	41.3	20.8	92.4	14	91.8	25.2
MEHHP	1	58.8	23.3	91.1	26.2	41.3	22.5	92.4	32.9	91.8	27.2
MEOHP	1	58.8	37.6	91.1	27.2	41.3	21.2	92.4	28.7	91.8	23

MECPP	1	58.8	16.1	91.1	26.6	41.3	18.9	92.4	23.9	91.8	24

groupPIP: group-specific posterior inclusion probability; condPIP: conditional posterior inclusion probability; BPA: bisphenol A; MBP: mono-n-butyl phthalate; MiBP: mono-isobutyl phthalate; MEP: monoethyl phthalate; MBzP: monobenzyl phthalate; MEHP: mono(2-ethylhexyl) phthalate; MEHHP: mono(2-ethyl-5-hydroxyhexyl) phthalate; MEOHP: mono(2-ethyl-5-oxohexyl) phthalate; MECPP: mono(2-ethyl-5-carboxypentyl) phthalate; HDL: high density lipoprotein; LDL: low-density lipoprotein. BKMR model was adjusted for age at sample collection, pre-pregnancy body mass index (BMI), education level, race, infertility diagnosis, mode of conception, number of fetuses, trimester and specific gravity. Hierarchical variable selection was done by grouping DEHP metabolites and BPA, parabens and other phthalate metabolites

Supplemental Table S5. Quantile g-computation measurement of the association between a quartile increase in mixture chemical biomarker concentrations and lipid biomarkers stratified by high vs low BMI.

		Mean difference	95% Confidence Interval
Low BMI	Triglyceride	0.01	-0.25 - 0.27
	Cholesterol	0.17	-0.09 - 0.42
	HDL	0.19	-0.18 - 0.57
	Non-HDL	0.13	-0.14 - 0.39
	LDL	0.15	-0.14 - 0.44
High BMI	Triglyceride	-0.02	-0.46 - 0.42
	Cholesterol	0.01	-0.45 - 0.47
	HDL	-0.36	-0.94 - 0.21
	Non-HDL	0.09	-0.38 - 0.57
	LDL	0.12	-0.43 - 0.67

BMI: body mass index; HDL: high-density lipoprotein; LDL: low-density lipoprotein.
Quantile g-computation models were adjusted for age at sample collection, education level, race, infertility diagnosis, mode of conception, number of fetuses, trimester and specific gravity.
